# A Pilot Safety Assessment for Recombinant *Epinephelus lanceolatus* Piscidin Yeast Powder as a Drug Food Additive after Subacute and Subchronic Administration to SD Rats

**DOI:** 10.3390/md18120586

**Published:** 2020-11-24

**Authors:** Bor-Chyuan Su, Chao-Chin Li, Chia-Wen Liu, Jyh-Yih Chen

**Affiliations:** 1 Department of Anatomy and Cell Biology, School of Medicine, College of Medicine, Taipei Medical University, Taipei 115, Taiwan; subc8265@tmu.edu.tw; 2 Institute of Cellular and Organismic Biology, Academia Sinica, Nankang, Taipei 115, Taiwan; jasper15@gate.sinica.edu.tw; 3 Marine Research Station, Institute of Cellular and Organismic Biology, Academia Sinica, 23-10 Dahuen Road, Jiaushi, Ilan 262, Taiwan; culex763@gmail.com

**Keywords:** recombinant *Epinephelus lanceolatus* piscidin, yeast powder, safety assessment, subacute toxicity, subchronic toxicity, SD rats

## Abstract

Recombinant *Epinephelus lanceolatus* piscidin (RELP) was previously shown to improve growth performance and immune response when used as a feed additive for *Gallus gallus domesticus*. However, the long-term toxicity of RELP has not be thoroughly investigated. In the present study, we evaluated the subacute and subchronic oral toxicities of RELP in SD rats by hematological, biochemical, and histopathological analyses. To determine subacute and subchronic toxicities, male and female rats were fed with RELP 1000 mg/kg bodyweight/day for 28 and 90 days, respectively. Bodyweight and food intake were unchanged by RELP treatment over the course of the studies. After exposure, samples of blood, heart, lung, liver, and kidney were collected and analyzed. Results demonstrated that RELP exposure did not cause any observable hematological, biochemical, or histological abnormalities in SD rats. Thus, RELP may be a safe feed additive for use in agriculture and aquaculture.

## 1. Introduction

Antibiotic resistance is a serious threat to global public health that is largely caused by inappropriate use of antibiotics in humans and livestock [1]. Thus, the World Health Organization strongly recommends that the use of antibiotics to prevent disease in healthy animals should be stopped in food animal agriculture. Currently, antibiotic feed additives are banned in Europe, USA, South Korea, Netherlands, and Taiwan [2]. Therefore, new functional feed additives may be alternatives to antibiotics for enhancing animal health, immunity, and growth performance [3].

In recent years, many studies have demonstrated the benefits of marine antimicrobial peptides in animal feed for increasing growth rate [4], enhancing immunity and antioxidant activity [5,6], and modulating gut microbiota [6]. Furthermore, we previously demonstrated that recombinant *Epinephelus lanceolatus* piscidin (RELP) exhibits bactericidal activity against Gram-positive and Gram-negative bacteria, and dietary of RELP supplementation improves growth performance and immune response in *Gallus gallus domesticus* [4]. In addition, RELP does not possess mutagenic activity or cause acute toxicity in cultured cells or mice [7]. However, the long-term toxicity of RELP remains unclear. Examination of long-term toxicity of RELP is a necessary prerequisite to any future application in livestock animals. In the present study, we evaluated the subacute and subchronic toxicities of RELP in SD rats through hematological, biochemical, and histopathological analyses alteration. Based on the lack of observed toxicity in all assays, we expect RELP may be useful as a feed additive in agriculture and/or aquaculture.

## 2. Results

### 2.1. Subacute Toxicity of RELP in SD Rats

**Figure 1:**
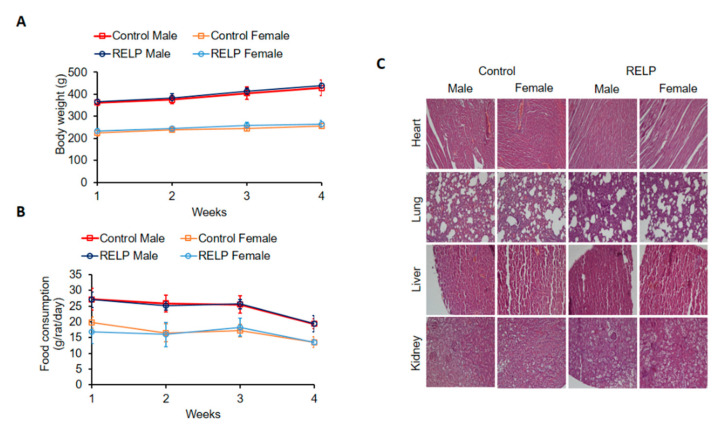
Recombinant *Epinephelus lanceolatus* piscidin (RELP) supplementation does not affect bodyweight, food intake, or histopathology of SD rats. SD rats were fed with control yeast powder or RELP yeast powder for 28 days. Bodyweight (**A**) and food intake (**B**) were monitored. Bodyweight: Control male vs. RELP male (*p* = 0.8668); control female vs. RELP female (*p* = 0.8385). Food consumption: Control male vs. RELP male (*p* = 0.9949); control female vs. RELP female (*p* = 0.9499). (**C**) Representative photomicrographs of H&E staining of heart, lung, liver, and kidney specimens from each group of rats (20× objective). Data represent mean ± SD. *n* = 5 per group.

SD rats were fed with RELP at a dose of 1000 mg/kg bodyweight/day for 28 days. Bodyweight and food consumption were measured at weekly intervals. RELP supplementation did not dramatically alter bodyweight in animals of either sex (Figure 1A). Furthermore, there were no differences in food intake between control and RELP-fed rats at any time point. However, food intake was slightly decreased at day 28 in control and RELP groups of both sexes (Figure 1B). Histopathological analysis demonstrated that there were no detectable histological abnormalities in any organ examined (heart, lung, liver, and kidney) after exposure to RELP (Figure 1C). We also assessed the final bodyweight (measured at day 28) and weight of each examined organ. Results demonstrated that neither final bodyweight nor organ weight were affected by feeding rats with RELP for both sexes (Table 1). Furthermore, hematological (WBC, NEU, LYM, MONO, EOS, BASO, RBC, HGB, HCT, MCV, MCH, MCHC, RDW, RET, PLT, MPV, PCT, and PDW; Table 2) and biochemical analyses (GOT, GPT, LDH, ALP, TBIL, TP, IP, BUN, CRE, UA, Ca, Mg, ALB, and NH_3_; Table 3) were performed. Although the measurements for WBC (*p* = 0.1097), NEU (*p* = 0.6760), MONO (*p* = 0.6495), and EOS (*p* = 0.4668) were relatively low in RELP-treated female rats, the differences were not significant when comparing control and RELP groups.

### 2.2. Subchronic Toxicity of RELP in SD Rats

**Figure 2:**
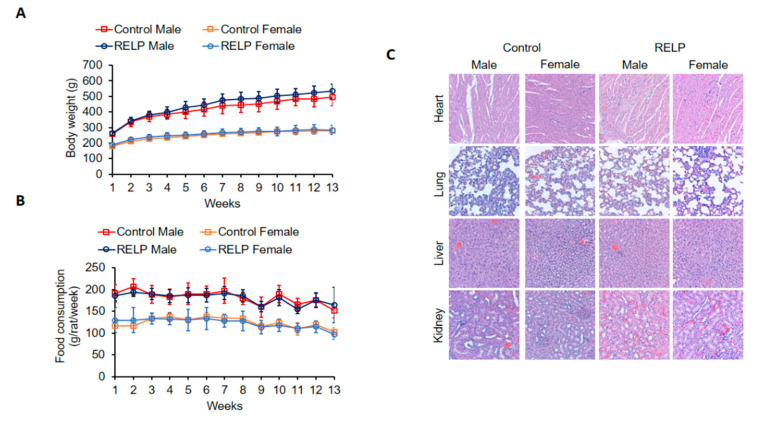
RELP supplementation does not affect bodyweight, food intake, or histology of SD rats. SD rats were fed with control yeast powder or RELP yeast powder for 90 days. Bodyweight (**A**) and food intake (**B**) were monitored. Bodyweight: Control male vs. RELP male (*p* = 0.4269); control female vs. RELP female (*p* = 0.9328). Food consumption: Control male vs. RELP male (*p* = 0.8734); control male vs. RELP male (*p* = 0.9777). (**C**) Representative photomicrographs of H&E staining of heart, lung, liver, and kidney specimens from each group rats (20× objective). Data represent mean ± SD. *n* = 4–5 per group.

To assess the subchronic toxicity, SD rats were fed with 1000 mg/kg bodyweight/day RELP for 90 days. Bodyweight exhibited a sustained increase during the treatment period in both groups of both sexes (Figure 2A). Food intake between RELP-fed and control groups was not significantly different for either sex (Figure 2B). Exposure to RELP did not cause any adverse histopathological features (Figure 2C). We also measured the final bodyweight (measured at day 90) and organ weights for lung, liver, heart, and kidney. Results showed no statistically significant difference between the control group and RELP-fed group for both sexes (Table 4). Hematological (Table 5) and biochemical (Table 6) parameters between diet groups were not significantly different for either sex.

## 3. Discussion

Antimicrobial peptides (AMPs) have been proposed as alternatives to antibiotics in livestock animal drug feed additives [8,9]. The peptides are known to enhance growth performance, promote nutrient utilization, and improve resistance to diseases [8,10], due to their bactericidal [11], immunomodulatory [11,12], and gut microbiota modulatory activities [6,13]. Marine organisms are one of rich sources of AMPs with promising beneficial activities [14]. We recently identified a new piscidin from giant grouper, *Epinephelus lanceolatus*, which possesses strong antibacterial activity against both Gram-positive and Gram-negative bacteria [4]. Oral administration of recombinant *Epinephelus lanceolatus* piscidin (RELP) improved growth performance and immunity in chickens [4], and short-term exposure to RELP did not induce any acute toxicity or mutagenicity in laboratory assays [7].

Toxicity tests for long-term exposure of a substance are essential to determine chemical safety [15]. Typical toxicity endpoints for long-term exposure studies are organ damage [16,17], tissue fibrosis [16,17], and hematological and biochemical parameters [15,18,19]. Subacute and subchronic toxicity assays are widely used to determine whether frequent exposure to a substance causes adverse effects [20]. Therefore, in order to better define the safety profile of RELP, SD rats were fed with RELP for 28 and 90 days; bodyweight, food intake, histopathology, and hematological and biochemical parameters were monitored.

Weight loss and reduction of food intake by test animals were considered abnormal. Neither subacute (Figure 1) nor subchronic (Figure 2) RELP supplementation caused bodyweight loss. Food intake was slightly decreased in rats fed with RELP for 28 days (Figure 1B); however, food intake was relatively consistent in rats fed with RELP for 90 days (Figure 2B), which means the minor reduction of food intake seen in the subacute study might have been due to normal fluctuations. Next, we examined whether RELP supplementation induced histopathological abnormalities. Four vital organs were examined, including heart, lung, liver, and kidney. Results showed that fibrosis or obvious tissue damage was absent in all observed organs. Minor organ injury might not be detected by histopathological examination, so we further analyzed biochemical parameters. Liver and kidney are the two major organs responsible for drug and toxin metabolism [21,22,23]. Glutamic oxaloacetic transaminase (GOT), glutamic pyruvic transaminase (GPT), total bilirubin (TBIL), albumin (ALB), alkaline phosphatase (ALP), and total protein (TP) were used to monitor liver function [24]. Blood urea nitrogen (BUN), creatinine (CRE), uric acid (UA), and ammonia (NH3) were measured to reflect kidney function [24]. Oral administration of RELP did not alter any kidney or liver indexes (Table 3 and Table 6), suggesting that RELP at dose of 1000 mg/kg bodyweight/day was not toxic to liver or kidney.

White blood cell (WBC) count reflects animal immunity [25]. We found that feeding female rats with RELP for 28 days slightly decreased the WBC number (Table 1). However, prolonged exposure to RELP (90 days) did not alter the WBC number in animals of either sex (Table 5). No other abnormalities were found in the hematological parameters after animals were fed with RELP for 28 and 90 days (Table 2 and Table 5).

In this study, our findings demonstrate that histological features, hematological endpoints, and biochemical parameters were not significantly affected by oral administration of RELP (1000 mg/kg bodyweight/day) to SD rats for 28 or 90 days. These results suggested a lack of toxicity from oral administration of RELP at doses up to 1000 mg/kg bodyweight/day in rats. Thus, the no-observed-adverse-effect level (NOAEL) values in rats appeared to be at least 1000 mg/kg bodyweight/day. Furthermore, according to the guidance on the assessment of the safety of feed additives for the target species [26], the maximum safe concentration [(NOAEL/100)/FI) × 1000 × 0.88] of crude recombinant EP powder in feed of chicken (FI = 0.158) and salmon (FI = 0.0021) could be up to 55.7 and 4190 g/g respectively. Thus, RELP may be a safe food additive, which could prove useful as a substitute for antibiotics in agriculture and aquaculture.

## 4. Materials and Methods

### 4.1. Recombinant Epinephelus Lanceolatus Piscidin Yeast Powder

Recombinant *Epinephelus lanceolatus* piscidin (RELP) was expressed in *Pichia pastoris* and fermented as previously described [4]. After fermentation, yeast cultures were collected and freeze-dried as yeast powder for the RELP group. For the control group, yeast cultures from pPICZ alpha (empty vector)-transformed *P. pastoris* X-33 were collected and processed in the same manner as the RELP yeast powder.

### 4.2. Animals and Experimental Design

Three-month-old Bltw SD rats were purchased from BioLASCO Taiwan Co., Ltd. (Taipei, Taiwan). Animals were housed in cages, according to sex. For the subacute toxicity experiment, 5 male and 5 female rats were assigned to the RELP group. Each rat was fed with RELP (1000 mg/kg bodyweight/day) for 28 days by intragastric gavage. RELP yeast powder was dissolved in distilled water. In the control group, animals (5 male and 5 female rats) were gavaged with the same amount of control yeast powder (pPICZ alpha-transformation). For the subchronic toxicity assay, there were 9 rats (5 males and 4 females) in both the control group and RELP group. Animals were fed with RELP (1000 mg/kg bodyweight/day) for 90 days in the same manner as the subacute (28-day) experiment. Bodyweight and food intake were measured weekly. At the indicated time-point, rats were sacrificed, and whole blood samples and organs (heart, liver, lung, and kidney) were collected. For hematological analysis, the following items were examined: White blood cells (WBC), neutrophils (NEU), lymphocytes (LYM), monocytes (MONO), eosinophils (EOS), basophils (BASO), red blood cells (RBC), hemoglobin (HGB), hematocrit (HCT), mean corpuscular volume (MCV), mean corpuscular hemoglobin (MCH), mean corpuscular hemoglobin concentration (MCHC), red cell distribution width (RDW), reticulocytes (RET), platelets (PLT), mean platelet volume (MPV), plateletcrit (PCT), and platelet distribution width (PDW). To analyze the biochemical parameters, serum was collected from each animal, and the following items were examined: Glutamic oxaloacetic transaminase (GOT), glutamic pyruvic transaminase (GPT), lactate dehydrogenase (LDH), alkaline phosphatase (ALP), total bilirubin (TBIL), total protein (TP), inorganic phosphorus (IP), blood urea nitrogen (BUN), creatinine (CRE), uric acid (UA), calcium (Ca), magnesium (Mg), albumin (ALB), and ammonia (NH_3_). Hematological and biochemical parameters were analyzed by the Taiwan Mouse Clinic (Taipei, Taiwan). To determine histopathological changes, formalin-fixed paraffin-embedded samples were sectioned and stained by hematoxylin and eosin (H&E). Histopathological features were examined by veterinarians at the Institute of Cellular and Organismic Biology Laboratory Animal Facility, Academia Sinica (Taipei, Taiwan). All procedures involving animals were conducted in accordance with the requirements of National Pingtung University of Science and Technology (NPUST), and were approved by the Animal Care and Use Committee of NPUST (NPUST-107-026).

### 4.3. Statistical Analysis

Data were expressed as mean ± standard deviation (SD). The differences in means of control and treatment groups were analyzed by one-way ANOVA, followed by Sidak’s multiple comparisons test. Statistical tests were conducted using GraphPad Prism software version 8.0 (GraphPad Software, San Diego, CA, USA). *p*-values less than 0.05 were considered statistically significant.

## 5. Conclusions

Our results showed that the oral administration of RELP did not alter bodyweight or organ weight of SD rats. Furthermore, RELP did not cause histopathological abnormalities or substantively affect hematological and biochemical parameters, regardless of animal sex. Thus, RELP might be a promising and safe feed additive.

## Figures and Tables

**Table 1:** Bodyweight and organ weight (lung, liver, heart, and kidney) of rats fed with or without RELP for 28 days.

	Male		Female	
	Control (*n* = 5)	RELP (*n* = 5)	Control (*n* = 5)	RELP (*n* = 5)
Mean bodyweight (g)	424.32 ± 29.95	422.34 ± 46.4	241.42 ± 10.59	253.94 ± 17.21
Lung (g)	1.6 ± 0.31	1.7 ± 0.14	1.2 ± 0.08	1.26 ± 0.22
Liver (g)	12.48 ± 1.66	13.16 ± 0.83	7.77 ± 1.3	7.89 ± 0.83
Heart (g)	1.64 ± 0.22	1.72 ± 0.2	1 ± 0.09	0.95 ± 0.11
Kidney (g)	2.14 ± 0.23	2.25 ± 0.32	1.28 ± 0.14	1.27 ± 0.19

Results are expressed as mean ± SD.

**Table 2:** Hematological parameters of rats fed with or without RELP for 28 days.

Parameters	Male		Female	
	Control (*n* = 5)	RELP (*n* = 5)	Control (*n* = 5)	RELP (*n* = 5)
WBC (K/μL)	8.99 ± 4.56	9.76 ± 5.46	11.34 ± 6.47	4.42 ± 1.15
NEU (K/μL)	0.84 ± 0.47	1.08 ± 0.85	0.79 ± 0.53	0.47 ± 0.28
LYM (K/μL)	7.72 ± 3.91	8.08 ± 4.62	10.15 ± 5.73	3.79 ± 1.01
MONO (K/μL)	0.35 ± 0.26	0.46 ± 0.41	0.29 ± 0.25	0.13 ± 0.07
EOS (K/μL)	0.07 ± 0.06	0.10 ± 0.11	0.08 ± 0.07	0.02 ± 0.01
BASO (K/μL)	0.02 ± 0.02	0.04 ± 0.03	0.03 ± 0.02	0.01 ± 0.01
RBC (M/μL)	9.37 ± 0.31	9.00 ± 0.74	8.69 ± 0.66	8.46 ± 0.52
HGB (g/dL)	17.14 ± 0.63	16.82 ± 1.40	15.9 ± 1.09	15.43 ± 0.84
HCT (%)	52.86 ± 1.30	50.66 ± 4.77	48.74 ± 3.99	46.75 ± 2.63
MCV (fL)	56.44 ± 0.84	56.26 ± 0.93	56.04 ± 1.11	55.28 ± 1.11
MCH (pg)	18.28 ± 0.44	18.72 ± 0.46	18.30 ± 0.53	18.25 ± 0.17
MCHC (g/dL)	32.42 ± 0.63	33.24 ± 0.76	32.64 ± 0.65	33.00 ± 0.35
RDW (%)	20.00 ± 0.39	19.70 ± 1.53	18.96 ± 0.83	18.98 ± 1.00
RET (K/μL)	240.12 ± 48.68	241.88 ± 41.57	273.66 ± 62.79	243.93 ± 13.40
PLT (10^9^/L)	812 ± 348.23	837.00 ± 161.20	1078.80 ± 176.70	920.25 ± 85.47
MPV (fL)	8.18 ± 0.19	8.52 ± 0.26	8.26 ± 0.34	8.63 ± 0.05
PCT (%)	0.77 ± 0.33	0.85 ± 0.16	0.90 ± 0.13	0.81 ± 0.08
PDW (fL)	9.32 ± 0.51	9.82 ± 0.30	8.60 ± 0.39	9.05 ± 0.34

Results are expressed as mean ± SD. WBC: White blood cells, NEU: Neutrophils, LYM: Lymphocytes, MONO: Monocytes, EOS: Eosinophils, BASO: Basophils, RBC: Red blood cells, HGB: Hemoglobin, HCT: Hematocrit, MCV: Mean corpuscular volume, MCH: Mean corpuscular hemoglobin, MCHC: Mean corpuscular hemoglobin concentration, RDW: Red cell distribution width, RET: Reticulocytes, PLT: Platelets, MPV: Mean platelet volume, PCT: Plateletcrit, and PDW: Platelet distribution width.

**Table 3:** Biochemical parameters for rats fed with or without RELP for 28 days.

Parameters	Male		Female	
	Control (*n* = 5)	RELP (*n* = 5)	Control (*n* = 5)	RELP (*n* = 5)
GOT (U/L)	96.40 ± 24.53	98.40 ± 36.25	78.40 ± 16.91	65.60 ± 14.94
GPT (U/L)	30.40 ± 3.91	50.60 ± 26.59	28.40 ± 6.88	29.80 ± 10.45
LDH (U/L)	452.80 ± 250.21	538.80 ± 85.53	217.60 ± 64.02	258.40 ± 107.16
ALP (U/L)	611.20 ± 186.06	513.00 ± 68.60	303.00 ± 110.77	278.40 ± 64.31
TBIL (mg/dL)	0.44 ± 0.15	0.70 ± 0.35	0.40 ± 0.7	0.36 ± 0.05
TP (g/dL)	6.64 ± 0.30	7.22 ± 1.51	7.46 ± 0.48	7.38 ± 0.65
IP (mg/dL)	11.08 ± 0.87	12.08 ± 1.54	9.32 ± 1.40	8.62 ± 0.58
BUN (mg/dL)	14.34 ± 2.09	16.10 ± 4.04	13.82 ± 3.15	14.16 ± 2.35
CRE (mg/dL)	0.36 ± 0.13	0.36 ± 0.15	0.24 ± 0.05	0.26 ± 0.09
UA (mg/dL)	2.38 ± 0.80	3.40 ± 1.47	1.98 ± 0.67	2.10 ± 0.68
Ca (mg/dL)	11.35 ± 0.72	11.92 ± 1.61	11.40 ± 0.55	11.40 ± 0.37
Mg (mg/dL)	3.54 ± 0.27	4.12 ± 0.75	3.28 ± 0.43	3.4 ± 0.25
ALB (g/dL)	4.60 ± 0.32	4.94 ± 0.55	5.14 ± 0.50	4.78 ± 0.24
NH_3_ (μg/dL)	187.60 ± 41.05	207.75 ± 46.80	147.60 ± 33.95	162.20 ± 31.09

Results are expressed as mean ± SD. GOT: Glutamic oxaloacetic transaminase, GPT: Glutamic pyruvic transaminase, LDH: Lactate dehydrogenase, ALP: Alkaline phosphatase, TBIL: Total bilirubin, TP: Total protein, IP: Inorganic phosphorus, BUN: Blood urea nitrogen, CRE: Creatinine, UA: Uric acid, Ca: Calcium, Mg: Magnesium, ALB: Albumin, and NH_3_: Ammonia.

**Table 4:** Bodyweight and organ weights (lung, liver, heart, and kidney) for rats fed with or without RELP for 90 days.

	Male		Female	
	Control (*n* = 5)	RELP (*n* = 5)	Control (*n* = 4)	RELP (*n* = 4)
Mean bodyweight (g)	496.32 ± 52.1	534.2 ± 41.8	278.8 ± 11.8	284.2 ± 28.4
Lung (g)	2 ± 0.2	2.1 ± 0.2	1.5 ± 0.1	1.6 ± 0.1
Liver (g)	15.9 ± 2.9	18.5 ± 2.3	9.4 ± 1	9.69 ± 0.9
Heart (g)	1.7 ± 0.3	1.7 ± 0.1	1.1 ± 0.1	1.1 ± 0.1
Kidney (g)	3.7 ± 0.5	4.1 ± 0.3	2.2 ± 0.2	2.2 ± 0.1

Results are expressed as mean ± SD.

**Table 5:** Hematological parameters of rats fed with or without RELP for 90 days.

Parameters	Male		Female	
	Control (*n* = 5)	RELP (*n* = 5)	Control (*n* = 4)	RELP (*n* = 4)
WBC (K/μL)	8.51 ± 2.81	6.47 ± 1.41	6.61 ± 1.93	6.14 ± 2.84
NEU (K/μL)	1.47 ± 0.79	1.09 ± 0.60	0.99 ± 0.46	1.22 ± 0.84
LYM (K/μL)	6.53 ± 2.47	4.37 ± 2.12	5.17 ± 1.38	4.59 ± 1.96
MONO (K/μL)	0.37 ± 0.14	0.19 ± 0.07	0.35 ± 0.18	0.23 ± 0.18
EOS (K/μL)	0.13 ± 0.06	0.15 ± 0.07	0.09 ± 0.02	0.10 ± 0.04
BASO (K/μL)	0.01 ± 0.01	0.01 ± 0.01	0.01 ± 0.01	0.01 ± 0.00
RBC (M/μL)	9.11 ± 0.73	9.22 ± 0.35	8.22 ± 0.27	7.91 ± 0.79
HGB (g/dL)	15.67 ± 1.02	15.43 ± 0.78	14.98 ± 0.46	14.13 ± 0.94
HCT (%)	50.26 ± 4.44	49.77 ± 2.38	46.44 ± 2.21	45.08 ± 4.00
MCV (fL)	55.16 ± 1.63	53.98 ± 2.08	56.50 ± 1.15	57.02 ± 0.93
MCH (pg)	17.23 ± 0.53	16.73 ± 0.59	18.24 ± 0.26	17.90 ± 0.71
MCHC (g/dL)	31.26 ± 0.81	31.00 ± 0.40	32.30 ± 0.89	31.40 ± 0.84
RDW (%)	21.04 ± 1.34	21.80 ± 0.67	17.94 ± 1.23	17.83 ± 1.87
RET (K/μL)	245.51 ± 39.99	230.62 ± 24.02	233.16 ± 26.69	244.77 ± 31.13
PLT (10^9^/L)	579.29 ± 330.14	769.67 ± 90.10	712.80 ± 138.34	612.50 ± 395.52
MPV (fL)	7.87 ± 1.10	7.62 ± 0.45	7.88 ± 0.40	7.63 ± 0.33
PCT (%)	0.46 ± 0.24	0.69 ± 0.04	0.60 ± 0.07	0.50 ± 0.28
PDW (fL)	7.71 ± 0.31	8.22 ± 0.57	7.90 ± 0.24	8.17 ± 0.51

Results are expressed as mean ± SD. WBC: White blood cells, NEU: Neutrophils, LYM: Lymphocytes, MONO: Monocytes, EOS: Eosinophils, BASO: Basophils, RBC: Red blood cells, HGB: Hemoglobin, HCT: Hematocrit, MCV: Mean corpuscular volume, MCH: Mean corpuscular hemoglobin, MCHC: Mean corpuscular hemoglobin concentration, RDW: Red cell distribution width, RET: Reticulocytes, PLT: Platelets, MPV: Mean platelet volume, PCT: Plateletcrit, and PDW: Platelet distribution width.

**Table 6:** Biochemical parameters for rats fed with or without RELP for 90 days.

Parameters	Male		Female	
	Control (*n* = 5)	RELP (*n* = 5)	Control (*n* = 4)	RELP (*n* = 4)
GOT (U/L)	145.57 ± 108.95	117.33 ± 44.03	81.60 ± 11.84	82.67 ± 25.93
GPT (U/L)	47.43 ± 21.49	41.50 ± 7.82	39.00 ± 10.34	36.83 ± 8.93
LDH (U/L)	1496.29 ± 1129.76	1496.67 ± 615.96	485.40 ± 431.91	947.67 ± 503.84
ALP (U/L)	930.71 ± 522.37	805.50 ± 95.69	322.80 ± 53.42	938.67 ± 511.45
TBIL (mg/dL)	0.53 ± 0.45	0.30 ± 0.09	0.28 ± 0.08	0.27 ± 0.08
TP (g/dL)	6.33 ± 0.49	6.20 ± 0.45	6.52 ± 0.29	6.55 ± 0.46
IP (mg/dL)	7.83 ± 1.43	8.07 ± 1.01	6.96 ± 0.85	7.63 ± 0.79
BUN (mg/dL)	17.37 ± 1.71	18.50 ± 2.43	16.56 ± 3.40	18.13 ± 1.59
CRE (mg/dL)	0.30 ± 0.06	0.31 ± 0.06	0.36 ± 0.11	0.31 ± 0.07
UA (mg/dL)	1.74 ± 0.44	2.38 ± 0.63	1.72 ± 0.40	1.52 ± 0.37
Ca (mg/dL)	9.77 ± 0.33	10.32 ± 0.67	10.88 ± 0.39	10.93 ± 1.12
Mg (mg/dL)	2.54 ± 0.36	3.28 ± 0.52	2.60 ± 0.19	2.98 ± 0.26
ALB (g/dL)	4.40 ± 0.61	4.30 ± 0.32	4.82 ± 0.52	4.95 ± 0.72
NH_3_ (μg/dL)	159.29 ± 68.63	220.17 ± 72.45	132.80 ± 39.18	155.83 ± 29.00

Results are expressed as mean ± SD. GOT: Glutamic oxaloacetic transaminase, GPT: Glutamic pyruvic transaminase, LDH: Lactate dehydrogenase, ALP: Alkaline phosphatase, TBIL: Total bilirubin, TP: Total protein, IP: Inorganic phosphorus, BUN: Blood urea nitrogen, CRE: Creatinine, UA: Uric acid, Ca: Calcium, Mg: Magnesium, ALB: Albumin, and NH_3_: Ammonia.
